# Intraoperative complications during gastrectomy for gastric cancer – incidence, treatment, and effect on postoperative complications and survival in a population-based nationwide study

**DOI:** 10.1007/s00423-025-03935-x

**Published:** 2025-11-26

**Authors:** Anna Junttila, Olli Helminen, Mika Helmiö, Heikki Huhta, Aapo Jalkanen, Raija Kallio, Vesa Koivukangas, Arto Kokkola, Simo Laine, Elina Lietzen, Johanna Louhimo, Sanna Meriläinen, Vesa-Matti Pohjanen, Tuomo Rantanen, Ari Ristimäki, Jari V. Räsänen, Juha Saarnio, Eero Sihvo, Vesa Toikkanen, Tuula Tyrväinen, Antti Valtola, Joonas H. Kauppila

**Affiliations:** 1 Department of Surgery, Kuopio University Hospital, Kuopio, Finland; 2 Surgery Research Unit, Medical Research Center Oulu, Oulu University Hospital, University of Oulu, Oulu, Finland; 3 Division of Digestive Surgery and Urology, Turku University Hospital, Turku, Finland; 4 Department of Surgery, University of Helsinki and Helsinki University Hospital, Helsinki, Finland; 5 Department of Oncology and Radiotherapy, Oulu University Hospital, Oulu, Finland; 6 Cancer and Translational Medicine Research Unit, Medical Research Center Oulu, University of Oulu and Oulu University Hospital, Oulu, Finland; 7 Department of Surgery, University of Eastern Finland, Kuopio University Hospital, Kuopio, Finland; 8 Department of Pathology, HUS Diagnostic Center, HUSLAB, Helsinki University Hospital, University of Helsinki, Helsinki, Finland; 9 Applied Tumor Genomics Research Program, Research Programs Unit, Faculty of Medicine, University of Helsinki, Helsinki, Finland; 10 Department of General Thoracic and Oesophageal Surgery, Heart and Lung Centre, University of Helsinki and Helsinki University Hospital, Helsinki, Finland; 11 Department of Surgery, Central Finland Central Hospital, Jyväskylä, Finland; 12 Department of Cardiothoracic Surgery, Heart Center, Tampere University Hospital, University of Tampere, Tampere, Finland; 13 Department of Gastroenterology and Alimentary Tract Surgery, Tampere University Hospital, Tampere, Finland; 14 Upper Gastrointestinal Surgery, Department of Molecular Medicine and Surgery, Karolinska Institutet and Karolinska University Hospital, Stockholm, Sweden

**Keywords:** Gastrectomy, Gastric adenocarcinoma, Intraoperative complication, Postoperative complication

## Abstract

**Purpose:**

Studies reporting intraoperative complications during gastrectomy for gastric cancer or their effect on short- and long-term outcomes are rare. Our aim was to examine the effect of major intraoperative complications to incidence of major postoperative complications and long-term survival after gastrectomy for gastric cancer.

**Methods:**

This population-based, nationwide, and retrospective cohort study reports intraoperative complications and examines the effect of major intraoperative complications to incidence of major postoperative complications and mortality after gastrectomy for gastric cancer in Finland in 2005–2016.

**Results:**

Total or partial gastrectomy was performed to 2,184 patients eligible for this study. A total of 552 (25.3%) intra-operative complications occurred in 483 patients. Major intraoperative complication occurred to 69 patients (3.2%) and major postoperative complication occurred to 374 patients (17.1%). The occurrence of major intraoperative complications was not associated to the risk of postoperative major complications in the crude (OR 1.13, CI 0.61–2.08) or in the adjusted analysis (OR 1.18, CI 0.62–2.27), compared to patients without major intraoperative complications. Major intraoperative complications were not associated to higher 90-day mortality (HR 1.76, 95% CI 0.81–3.82) or higher 5-year mortality (HR 1.09, 95% CI 0.79–1.52) compared to patients without major intraoperative complications.

**Conclusion:**

Intraoperative complications during gastric cancer surgery are common but mainly not life-threatening and can be managed with relatively low sequelae. Major intraoperative complications did not increase the risk of major postoperative complications and were not associated to higher 90-day, or 5-year mortality compared to patients without major intraoperative complications after gastric cancer surgery.

## Introduction

Gastric cancer is one of the leading causes of cancer-related death worldwide [1]. The only curative treatment of gastric cancer is surgical resection with or without perioperative chemotherapy [2]. Studies reporting intraoperative complications after gastric cancer surgery are rare while the postoperative complication rates reported after gastric cancer surgery are high ranging from 9% to 46% and there are studies showing that complications are related to poorer overall survival [3–6].

An international, multicentre cohort study of 2,520 patients having any type of surgery found that up to 24% of patients experience at least one intraoperative complication and higher grade of intraoperative complications increased the risk for more severe postoperative complications [7]. A German comprehensive analysis (2008–2018) of 63,619 patients undergoing subtotal or total gastrectomy for gastric cancer found 4,035 patients (6.3%) with intraoperative injury to vessels or organ or bleeding complication [8]. The rate of intraoperative complications varied from 2.5% to 10.4% and was comparable between laparoscopic and open gastrectomy in the systematic review and meta-analysis [9] of seven randomized controlled trials [10–16]. However, the effect of intraoperative complications on the incidence of postoperative complications or survival after gastric cancer surgery are somehow unclear. A meta-analysis of 4,653 patients showed that larger intraoperative bleeding increased the rate of postoperative complications, and decreased both overall and disease-free survival of gastric cancer patients [17]. A retrospective post-hoc analysis (*n* = 528) of the FUGES-001 [18] and CLASS-01 [10] studies found that the overall, disease-specific, and recurrence-free survival were significantly better in the non-intraoperative complication group than in intraoperative complication group after laparoscopic radical surgery for gastric cancer [19]. Further analysis in the same study found that advanced tumor stage was an independent risk factor for poorer prognosis and increased the risk of intraoperative complications [19].

There is a lack of extensive analysis of intraoperative complications during total or partial gastrectomy for gastric cancer. This Finnish study reports intraoperative complications of gastric cancer surgery in population-based, nationwide setting. Furthermore, the aim of the present study was to examine the effect of major intraoperative complications to incidence of major postoperative complications and long-term survival after gastrectomy for gastric cancer in Finnish population-based nationwide study.

## Materials and methods

### Study design

This was a population-based, nationwide, and retrospective cohort study from Finland including all patients undergoing total or partial gastrectomy for gastric adenocarcinoma. All the other resection types or patients with completely missing patient records or unclear exposure information were excluded. Patients with other histological types than gastric adenocarcinoma were excluded because they are not comparable in terms of treatment. The study period was from 1 January 2005 to 31 December 2016, with follow-up until 31 December 2019 [20]. The study was approved by the Regional Ethical Review Board in Oulu, Finland, the Finnish national health officials and hospital districts [21].

### Data collection

The Finnish National Esophago-Gastric Cancer Cohort (FINEGO) includes all patients with esophageal and gastric cancer diagnosed in Finland between 1987 and 2016 [20]. FINEGO database unifies information from the Finnish Cancer Registry, Finnish National Institute for Health and Welfare registries, Care Register for Healthcare and Hospital Discharge Registry, highly complete for gastric cancer [22]. Surgically treated patients were identified using NOMESCO surgical codes. The identification using both registries by searching for cancer diagnoses and operation codes allows near 100% completeness on eligible patient identification. After identification of cases, available information including age, sex, comorbidity [23], surgery, and other variables were collected from the Finnish Cancer Registry, Finnish National Institute for Health and Welfare registries, Care Register for Healthcare and Hospital Discharge Registry [20]. Postoperative all-cause mortality data was obtained from the 100% complete death registry, held by Statistics Finland until 31st December 2019 [24].

Medical reports were obtained from the respective healthcare units, and reviewed by specialized gastrointestinal surgeons, providing accurate information on tumor location, histology, stage, neoadjuvant treatment, type of resection and adjuvant therapy. Data of intra-operative complications was collected separately. Intraoperative complications were classified according to Definition and Classification of Intraoperative Complications (CLASSIC) into grade 0-IV (grade 0 no deviation from the ideal intraoperative course, grade I any deviation from the ideal intraoperative course, without need for additional treatment or intervention, grade II any deviation from the ideal intraoperative course requiring treatment or intervention, but not leading to disability and not life-threatening, grade III any deviation from the ideal intraoperative course requiring treatment or intervention, life-threatening or leading to permanent disability, and, grade IV any deviation from the ideal intraoperative course leading to death of the patient) [25]. Furthermore, Classic categorizes grade I–II complications as minor complications and grade III–IV life-threatening complications as major complications and according to this, Classic grade III and above complications were considered as major intraoperative complications. Additional organ resections due to oncological reasons were not considered as intraoperative complications. Data of post-operative complications was also collected separately. Postoperative complications were classified according to the Clavien-Dindo classification into grade 0-V [26]. Clavien-Dindo grade IIIa and above complications were considered as major postoperative complications.

### Outcomes

The primary outcome was 90-day major postoperative complications, pulmonary complications, cardiac complications, gastrointestinal complications, and infectious complications, as defined by the ECCG classification [27] in gastric cancer surgery.

The secondary outcome was to evaluate overall 90-day and 5-year survival of gastric adenocarcinoma patients undergoing total or partial gastrectomy with no major intraoperative complications or with major intraoperative complications.

### Statistical analysis

The analyses of the present study followed a detailed a priori study protocol. IBM SPSS v26.0 (IBM Corp., Armonk, NY) was used for all analyses. Descriptive tables were produced. Follow-up times were calculated from the date of surgery until the time of death or the end of follow-up, whichever occurred first. Survival was calculated using the life table method, visualized with Kaplan-Meier curves. Logistic regression provided odds ratios (OR) and Cox regression provided hazard ratios (HR) with 95% confidence intervals (CI). To avoid confounding adjustments for ten known prognostic factors were made: age (continuous), sex (male/female), year of the surgery (continuous), comorbidity (according to Charlson Comorbidity Index [23] 0, 1 or ≥ 2 (excluding the gastric cancer under treatment)), histology (diffuse, intestinal or mixed adenocarcinoma, according to Lauren Classification [28]), tumor location (proximal, middle, distal), resection type (total, distal or proximal), radicality (R0, R1, R2 or palliative resection), pathological stage (stage 0-I, II, III, IV, according to 8th edition AJCC/UICC staging of gastric cancer [29]), and neoadjuvant therapy (yes/no). Patients with completely missing exposure or outcome data were excluded from the analysis.

## Results

### Patients

During 2005–2016 total or partial gastrectomy was performed to 2,196 patients. Twelve patients with missing exposure or primary outcome data were excluded and the final analysis included 2,184 gastric adenocarcinoma patients. The most common procedure was total gastrectomy which was performed to 1,318 patients (60.3%). The median age of all patients was 71 years. Majority of the patients had pathological stage III disease. Patient characteristics are described in Table 1.

**Table 1:** Clinical variables in 2,184 gastrectomy patients operated for gastric adenocarcinoma in Finland 2005 to 2016

	No major intraoperative complication *n* = 2,115	Major intraoperative complication *n* = 69
Sex, *n* (%)		
Female	932 (44.1)	31 (44.9)
Male	1,183 (55.9)	38 (55.1)
Charlson comorbidity index, n (%)		
0	1,067 (50.4)	33 (47.8)
1	636 (30.1)	23 (33.3)
2	253 12.0)	7 (10.1)
3	159 (7.5)	6 (8.7)
Tumor location, n (%)		
Proximal (including cardia)	259 (12.3)	9 (13.0)
Middle	878 (41.5)	36 (52.2)
Distal	977 (46.2)	24 (34.8)
Neoadjuvant treatment, n (%)		
No	1,824 (86.3)	55 (80.9)
Yes	287 (13.6)	12 (17.6)
Missing	3 (0.1)	1 (1.5)
Pathological stage, n (%)		
0-I	513 (24.7)	16 (23.9)
II	605 (29.1)	24 (35.8)
III	734 (35.3)	24 35.8)
IV	225 (10.8)	3 (4.5)
Lauren Classification		
Diffuse	1,005 (47.5)	32 (46.4)
Intestinal	945 (44.7)	29 (42.0)
Indeterminate	115 (5.4)	6 (8.7)
Unclear	50 (2.4)	2 (2.9)
Resection type		
Total gastrectomy	1,260 (59.6)	58 (84.1)
Proximal gastrectomy	24 (1.1)	0
Distal gastrectomy	825 (39.0)	11 (15.9)
Other	6 (0.3)	0
Operative approach		
Open	2,018 (95.4)	63 (91.3)
Laparoscopic	97 (4.6)	6 (8.7)
Lymphadenectomy		
D0	319 (15.1)	4 (5.8)
D1	992 (46.9)	32 (46.4)
D2	744 (35.1)	32 (46.4)
Missing	60 (2.8)	1 (1.4)
Radicality		
R0	1,531 (75.2)	51 (78.5)
R1	170 (8.4)	7 (10.8)
R2	160 (7.9)	4 (6.2)
Palliative intent	174 (8.6)	3 (4.6)
Adjuvant treatment		
Yes	985 (50.1)	27 (42.9)
No	981 (49.9)	36 (57.1)
Postoperative complications (Clavien-Dindo Classification)		
No complication or Grade I	1,210 (57.2)	38 (55.1)
Grade II	544 (25.7)	18 (26.1)
Grade III	211 (10.0)	7 (10.1)
Grade IV	90 (4.3)	2 (2.9)
Grade V (in-hospital mortality)	60 (2.8)	4 (5.8)
90-day complications		
Any 90-day complication	910 (43.0)	32 (46.4)
Major 90-day complication	361 (17.1)	13 (18.8)
Pulmonary 90-day complication	320 (15.1)	14 (20.3)
Cardiac 90-day complication	150 (7.1)	5 (7.2)
Gastrointestinal 90-day complication	417 (19.7)	17 (24.6)
Urologic 90-day complication	95 (4.5)	5 (7.2)
Thromboembolic 90-day complication	46 (2.2)	0
Neurologic 90-day complication	52 (2.5)	2 (2.9)
Infectious 90-day complication	357 (16.9)	18 (26.1)
Wound 90-day complication	44 (2.1)	1 (1.4)
Other 90-day complication	48 (2.3)	2 (2.9)

### Intraoperative complications

Altogether 552 complications were reported from 483 patients. Of these, 423 (87.6%) patients had one intraoperative complication, 53 (11.0%) patients had two intraoperative complications and 7 (1.4%) patients had three or more intraoperative complications. Minor intraoperative complication occurred to 414 patients (19.0%) and major (Classic ≥ III) intraoperative complication to 69 patients (3.2%). The three most common complications were splenic injury without need for splenectomy *n* = 185 (33.5%), vascular injury *n* = 69 (12.5%) and splenic injury requiring splenectomy *n* = 61 (11.1%). These three most common complications presented 57.3% of all complication types. Of intraoperative injuries 72 organ injuries led to organ resection or removal. No intraoperative deaths existed. Detailed intraoperative complication data is presented in Table 2.

**Table 2:** Description of all the intraoperative complications

Intraoperative complication *n* (%)	*n* = 552
Splenic injury without splenectomy	185 (33.5)
Vascular injury	69 (12.5)
Splenic injury with splenectomy	61 (11.1)
Pancreatic injury	51 (9.2)
Liver injury	44 (8.0)
Diffuse bleeding	39 (7.1)
Small intestine injury	30 (5.4)
Anastomotic complication and re-anastomosis	24 (4.3)
Large intestine injury	23 (4.2)
Duodenal injury	8 (1.4)
Bile leak	5 (0.9)
Thoracic duct injury	4 (0.7)
Common bile duct injury	3 (0.5)
Gastric injury	2 (0.4)
Esophageal injury	2 (0.4)
Pleural injury	2 (0.4)

**Fig. 1:**
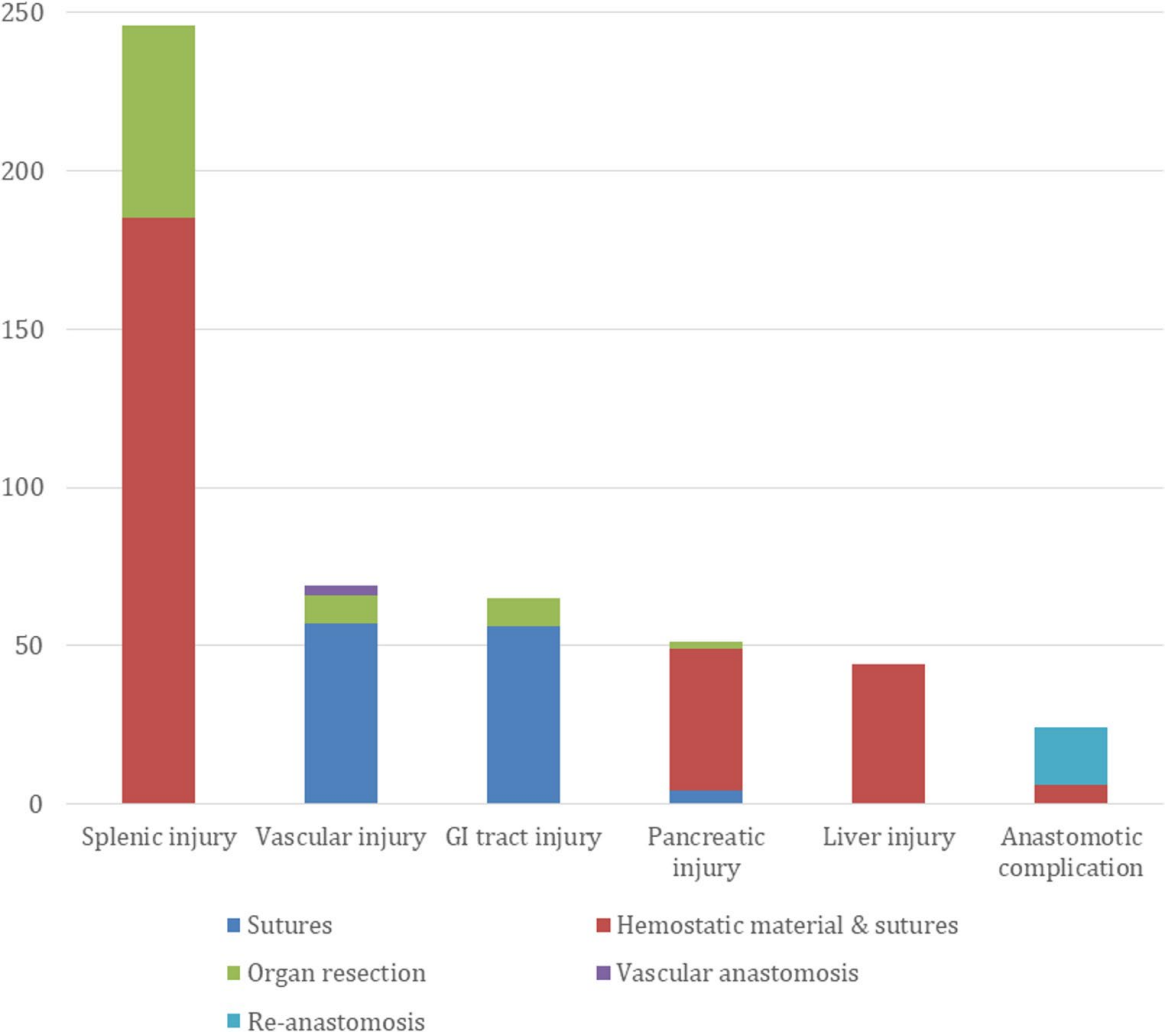
Intraoperative injury types according to their management

Injury types according to their management are presented in Fig. 1.

Of splenic injuries, 185 (75.2%) were minor and 61 (24.8%) major injuries. Minor splenic injuries were managed with compression, commercial hemostatic material, sutures or clips, or combining aforementioned methods. Major injuries to spleen causing massive or continuing bleeding were managed with splenectomy.

Of vascular injuries, 31 (44.9%) were arterial injuries, 20 (29.0%) were venous injuries and 18 (26.1%) were combined vascular injuries. Of all vascular injuries, nine (13.0%) lead to organ injury / failure and removal. Of these, lienal artery injuries lead to splenectomy in five patients, vascular injury to the mesenterium of jejunum lead to jejunum resection in two patients, and vascular injury to the mesenterium of colon lead to colon resection in two patients. In three (4.3%) vascular injuries vascular anastomosis was made by vascular surgeon. Of these, one right hepatic artery injury was caused by stapling the artery with duodenum, one injury to right hepatic artery and portal vein were caused due to preparation in challenging circumstances, and in one patient in control of massive bleeding right hepatic artery was sutured and vascular re-anastomosis was made with flap from gastroduodenal artery. Diffuse bleeding was managed with combining compression, sutures, and commercial hemostatic material.

All of the liver injuries were managed with compression, commercial hemostatic material, sutures, or combining aforementioned methods. Bile leaks from gallbladder resection site were managed with sutures and commercial hemostatic material. One common bile duct injury due to stapler injury was managed with stent and sutures, one injury to right hepatic duct was managed with hepaticojejunostomy and one bile leak after cholangiography was managed with sutures.

Majority of the pancreatic parenchymal injuries and all of the pancreatic vascular injuries were managed with sutures or commercial hemostatic material. Four (7.8%) parenchymal injuries were managed with sutures combined with omentum patch and two (3.9%) patients needed distal pancreatectomy.

Technical problems with anastomosis occurred in 24 (4.3%) patients. Of these, re-anastomosis was required in 17 (70.8%) patients in which one needed re-resection to esophagus and one to small intestine. Seven re-anastomosis was made due to rotation of the anastomosis and six re-anastomosis were required due to technical problems with stapler causing tear or hole to anastomosis. One anastomosis was made to biliary limb and one to distal ileum and therefore required re-anastomosis. Two patients needed distal re-anastomosis due to tear in the anastomosis. Sutures were applied to six (25%) anastomosis of which one due to bleeding and commercial hemostatic material was applied to one anastomosis.

Esophageal, gastric, and duodenal injuries were managed with sutures, or the damaged part was resected with the specimen. Six (20.0%) of the small bowel injuries and three (13.0%) of the large bowel injuries required bowel resection.

Thoracic duct injuries were managed with sutures and commercial hemostatic material. One tension pneumothorax was managed with intraoperative drainage and one pleural defect was sutured.

### Primary outcomes

Postoperative Clavien-Dindo II-V complication occurred to 936 (42.9%) patients and major (Clavien-Dindo ≥ IIIa) postoperative complication to 374 patients (17.1%).

The incidence of any postoperative complications was 46.4% and major postoperative complications 18.8% in those with major intraoperative complications and 43.0% and 17.1% respectively among those without major intraoperative complications (Table 1). Majority of the postoperative complications were pulmonary (20.3% vs. 15.1%), gastrointestinal (24.6% vs. 19.7%), and infectious complications (26.1% vs. 16.9%), among those with and without major intraoperative complications, respectively (Table 1). Detailed distribution of all the 90-day postoperative complications is presented in Table 3.

**Table 3:** Detailed description of all the 90-day postoperative complications. Classic grade III and above complications were considered as major intraoperative complications and Clavien-Dindo grade IIIa and above complications were considered as major postoperative complications

	No major intraoperative complication *n* = 2,115	Major intraoperative complication *n* = 69
Pulmonary	320 (15.1)	14 (20.3)
Pneumonia	247	11
Pleural effusion	100	5
Pneumothorax	6	0
Atelectasis	7	0
Respiratory insufficiency	36	1
ARDS	14	1
Aspiration	18	1
Cardiac	150 (7.1)	5 (7.2)
Cardiac arrest	21	0
Cardiac insufficiency	33	1
Atrial fibrillation	66	3
Ventricular fibrillation	4	0
Congestive heart failure	64	3
Pericarditis	1	0
Gastrointestinal	417 (19.7)	17 (24.6)
Anastomotic complication	102	4
Anastomotic leakage type 1	23	0
Anastomotic leakage type 2	19	0
Anastomotic leakage type 3	60	4
Ileus	104	2
Small bowel obstruction	22	0
J-tube complication	10	0
Clostridium difficile infection	16	0
Bleeding	80	4
Delayed emptying	36	1
Pancreatitis	10	0
Pancreatic fistula	14	1
Liver dysfunction	19	5
Biliary leakage	9	2
Splenic infarction	15	0
Urologic	95 (4.5)	5 (7.2)
Acute renal failure	26	2
Dialysis	7	1
Urinary tract infection	47	2
Retention	23	1
Thromboembolic	46 (2.2)	0
Deep venous thrombosis	8	0
Pulmonary embolus	30	0
Stroke	10	0
Neurologic	52 (2.5)	2 (2.9)
Recurrent nerve palsy type 1	1	0
Other neurologic injury	16	1
Delirium	37	1
Infectious	357 (16.9)	18 (26.1)
Wound infection	58	3
Central iv line infection	12	0
ntra-abdominal abscess	160	7
Intrathoracic abscess.	7	2
Sepsis	51	4
Other	128	6
Wound	44 (2.1)	1 (1.4)
Abdominal wound dehiscence	41	1
Acute abdominal wall hernia	2	0
Diaphragmatic hernia	1	0
Other	48 (2.3)	2 (2.9)
Chyle leakage type 1	12	0
Chyle leakage type 2	4	0
Chyle leakage type 3	1	0
Re-operation	17	0
Multi organ failure	16	2

The occurrence of major intraoperative complications was not associated to the risk of postoperative major complications in the crude (OR 1.13, CI 0.61–2.08) or in the adjusted analysis (OR 1.18, CI 0.62–2.27), compared to patients without major intraoperative complications (Table 4). The major intraoperative complications were associated to higher risk of 90-day infectious complications in the crude model (OR 1.74, CI 1.00-3.01) while there was no statistical significance in the adjusted model (OR 1.71, CI 0.95–3.09) compared to patients without intraoperative complications (Table 4).

**Table 4:** 90-day postoperative complications with no major or major intraoperative complications during gastrectomy for gastric adenocarcinoma, expressed as odds ratios (OR) with 95% confidence intervals (CI). Classic grade III and above complications were considered as major intraoperative complications and Clavien-Dindo grade IIIa and above complications were considered as major postoperative complications

	Number of the patients	No major intraoperative complication	Major intraoperative complication
Major postoperative complication			
All patients (crude)	2,184	1.00 (Reference)	1.13 (0.61–2.08)
All patients (adjusted)*	2,184	1.00 (Reference)	1.18 (0.62–2.27)
Pulmonary complication			
All patients (crude)	2,184	1.00 (Reference)	1.43 (0.79–2.60)
All patients (adjusted)*	2,184	1.00 (Reference)	1.41 (0.75–2.67)
Cardiac complication			
All patients (crude)	2,184	1.00 (Reference)	1.02 (0.41–2.58)
All patients (adjusted)*	2,184	1.00 (Reference)	0.91 (0.31–2.62)
Gastrointestinal complication			
All patients (crude)	2,184	1.00 (Reference)	1.33 (0.76–2.33)
All patients (adjusted)*	2,184	1.00 (Reference)	1.47 (0.82–2.66)
Infectious complication			
All patients (crude)	2,184	1.00 (Reference)	1.74 (1.00-3.01)
All patients (adjusted)*	2,184	1.00 (Reference)	1.71 (0.95–3.09)

^*^ Adjustment for age (continuous), sex (male/female), year of the surgery (continuous), CCI (0, 1 or ≥ 2 (excluding the gastric cancer under treatment)), histology (diffuse, intestinal or mixed adenocarcinoma), tumor location (proximal, middle, distal), resection type (total, distal or proximal), radicality (R0, R1, R2 or palliative resection), pathological stage (stage 0-I, II, III, IV, according to 8th edition AJCC/UICC staging of gastric cancer), and neoadjuvant therapy (yes/no)

### Secondary outcomes

The observed 90-day survival was 88.4% in patients with major intraoperative complications and 95.8% in patients without major intraoperative complications. Major intraoperative complications were not associated to higher 90-day mortality (HR 1.76, 95% CI 0.81–3.82, Table 5) compared to patients without major intraoperative complications after adjustment for confounders.

**Table 5:** 90-day and 5-year mortality (expressed as hazard ratios with 95% confidence intervals) and cancelled adjuvant therapy (expressed as odds ratios with 95% confidence intervals) without major or with major intraoperative complication during gastrectomy for gastric adenocarcinoma

	Number of the patients	No major intraoperative complication	Major intraoperative complication
90-day mortality			
All patients (crude)	2,184	1.00 (Reference)	1.67 (0.82–3.40)
All patients (adjusted)*	2,184	1.00 (Reference)	1.76 (0.81–3.82)
5-year mortality			
All patients (crude)	2,184	1.00 (Reference)	1.00 (0.73–1.36)
All patients (adjusted)*	2,184	1.00 (Reference)	1.09 (0.79–1.52)
Cancelled adjuvant therapy			
All patients (crude)	2,184	1.00 (Reference)	1.34 (0.81–2.22)
All patients (adjusted)*	2,184	1.00 (Reference)	2.00 (1.05–3.80)

^*^ Adjustment for age (continuous), sex (male/female), year of the surgery (continuous), CCI (0, 1 or ≥ 2 (excluding the gastric cancer under treatment)), histology (diffuse, intestinal or mixed adenocarcinoma), tumor location (proximal, middle, distal), resection type (total, distal or proximal), radicality (R0, R1, R2 or palliative resection), pathological stage (stage 0-I, II, III, IV, according to 8th edition AJCC/UICC staging of gastric cancer), and neoadjuvant therapy (yes/no)

**Fig. 2:**
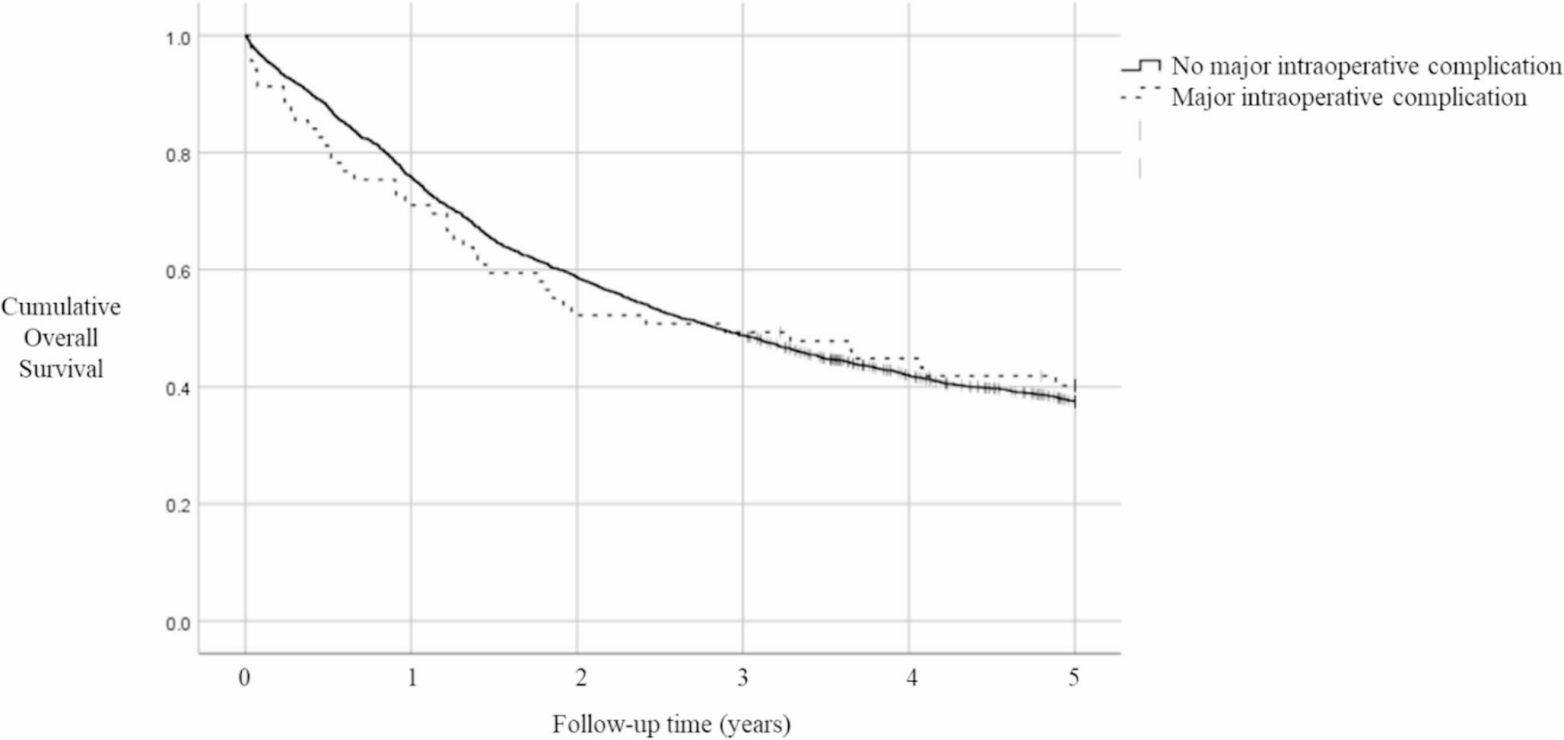
The observed overall 5-year survival in patients with no major intraoperative complication and in patients with major intraoperative complication

The observed 5-year survival was 40.2% in patients with major intraoperative complications and 37.5% in patients without major intraoperative complications (Fig. 2). Major intraoperative complications were not associated to higher 5-year mortality (HR 1.09, 95% CI 0.79–1.52) compared to patients without major intraoperative complications (Table 5).

Adjuvant treatment was started to 42.9% patients in those with major intraoperative complications and to 50.1% patients in those without major intraoperative complications. Major intraoperative complications were associated to higher risk of planned but cancelled adjuvant therapy (OR 2.00, CI 1.05–3.80) compared to patients without major intraoperative complications.

## Discussion

This population-based, nationwide study found that major intraoperative complications are rare and do not increase the risk of major postoperative complications after gastric cancer surgery. Major intraoperative complications were not associated to higher 90-day mortality, or 5-year mortality compared to patients with no major intraoperative complications.

The main strength of the present study is the population-based, nationwide study setting including all the patients diagnosed with gastric cancer undergoing total or partial gastrectomy with 100% complete follow-up in Finland. The Finnish national registries are based on automatic and independent reporting of diagnose and procedure codes from the hospitals to hospital discharge registry and also clinicians reporting new cancer cases enabling dependable patient identification with high coverage [22]. The large sample size of the cohort (*n* = 2,184) and the low percentage (0.5%) of excluded patients increases the quality of the present study. The intra- and postoperative complication data was comprehensively collected and categorized by specialized surgeons according to standardized classifications (the CLASSIC classification and the Clavien-Dindo classification) enabling future comparisons with other studies. This study has some limitations too. Since this study was a retrospective study, there is a possibility that some complications may have been missed during the review of patient records or some minor intraoperative complications may not have been reported by operating surgeon into patient records. Even though the results were adjusted for potential confounders some unknown confounding or bias may have occurred due to observational nature of the study. It is also noteworthy that most of the study patients underwent an open gastrectomy, whereas nowadays a large proportion of gastric cancer patients undergo minimally invasive gastrectomy. It can therefore be speculated whether, with the advancement of minimally invasive techniques and progress along the learning curve, the rate of intraoperative complications is nowadays lower than before. However, this is the first study investigating the effect of major intraoperative complications to incidence of postoperative complications and mortality after total or partial gastrectomy for gastric cancer in population-based nationwide setting.

Studies reporting intraoperative complications after gastric cancer surgery are rare. A comprehensive analysis of 63,619 patients undergoing subtotal or total gastrectomy for gastric cancer found 4,035 patients (6.3%) with intraoperative injury to vessels or organ or bleeding complication [8]. The largest systematic review and meta-analysis of randomized controlled trials found that the rate of intraoperative complications varied from 2.5% to 10.4% in different studies [10–16] and was comparable between laparoscopic and open gastrectomy in the meta-analysis [9]. In addition, laparoscopic gastrectomy has been shown to be associated with less intraoperative blood loss compared to open gastrectomy [11, 30, 31]. In our study the rate of all intraoperative complications was 22.1% during total or partial gastrectomy while the rate of major complications was 3.2%. High rate of intraoperative complications in our series may be due to strict reporting of intraoperative complications, which is seen as a high rate of minor bleeding complications requiring relatively simple treatment, for example application of commercial hemostatic material on the bleeding. Our unselected patient cohort included also palliative and emergency gastrectomies where patients are not preoperatively optimized to major gastric cancer surgery. Furthermore, patients were operated in both low and high-volume centers. However, the rate of major complications was low and comparable to studies published before.

Gastrectomy is associated with a high risk of postoperative complications and even rates up to 37.4% have been reported [6]. Most frequent postoperative complications after gastric cancer surgery are pulmonary complications, anastomotic leakage, and wound complications [32]. Anastomotic leakage and pulmonary complications have been demonstrated to have the greatest overall impact on postoperative mortality, re-interventions, and reoperations whereas intra-abdominal abscesses and wound infections also have a high impact on re-interventions, reoperations, and hospital readmissions [32]. The study of 9,288 patients undergoing abdominal surgery found that patients with intraoperative complications had higher risk of developing postoperative complications especially deep/organ-space surgical site infection, sepsis and pneumonia [33]. In addition, the sensitivity analysis of 1,440 patients undergoing gastrectomy had higher rates of 30-day morbidity and deep/organ-space surgical site infection [33]. A pooled analysis of two randomized controlled trials of 528 gastric cancer patients found that the rate of postoperative and severe postoperative complications was higher in patients with intraoperative complications compared to those without intraoperative complications [19]. Furthermore, intraoperative complications are known to cause delays in adjuvant therapy and higher grade of intraoperative complications is associated with more severe postoperative complications [19, 33, 34]. In this current population-based nationwide cohort study the incidence of major intraoperative complications during total or partial gastrectomy was 3.2% which is in line with earlier published data [10–16]. The incidence of major postoperative complications was 18.8% in those with major intraoperative complications and 17.1% in those without major intraoperative complications. The incidence of the most common postoperative complications (pulmonary, gastrointestinal, and infectious complications) was higher in those with major intraoperative complications compared to those without major intraoperative complications. The risk of infectious postoperative complications was over 1.7-fold in patients with major intraoperative complications compared to those without major intraoperative complications, but the finding was not statistically significant in the adjusted analysis. Intraoperative complications may reflect technically demanding procedures and advanced stage of gastric cancer, while their low number, coupled with the fact that some even major intraoperative complications can be managed without major deviation from the planned procedure, may reduce statistical power and explain why no significant effect was observed in the adjusted analysis. Also in our study, major intraoperative complications were associated to higher risk of planned but cancelled adjuvant therapy compared to patients without major intraoperative complications.

The 90-day mortality for gastrectomy due to gastric cancer has varied from 7.2% to 16.7% in earlier studies [35, 36]. In our study the 90-day mortality was 11.6% in patients with major intraoperative complications and 4.2% in patients without major intraoperative complications. The study of 9,288 patients undergoing abdominal surgery found that the risk of 30-day mortality was increased in patients with intraoperative complications compared to those without intraoperative complications and the increase was even higher in patients with major intraoperative complications [33]. This finding was also verified in the sensitivity analyses of 1,440 gastrectomy patients [33]. In our study, the risk estimate of 90-day mortality was higher in patients with major intraoperative complications compared to those without major intraoperative complications but without statistical significance. As earlier described the incidence of some postoperative complications was more frequent in patients with major intraoperative complications in our data. Patients undergoing gastric cancer surgery are often old, have multiple comorbidities and preoperative malnutrition and major intra- and postoperative complications are likely to reduce physical function and recovery as well as increase 90-day mortality. A study of 1,107 radically resected gastric cancer patients found that postoperative complications influenced overall survival due to complication-related early postoperative deaths but if patients successfully recovered from early postoperative complications major postoperative complications were not risk factors for decreased survival [37].

Studies of the effect of the intraoperative complications to long-term survival after gastric cancer surgery are very few. Intraoperative complications have been speculated to relate long-term outcomes especially due to higher risk of severe postoperative complications [7, 19]. A pooled analysis from two randomized trials found the overall, disease specific, and recurrence free survivals were significantly better in patients with no intraoperative complications compared to patients with intraoperative complications but in further analysis advanced tumor stage was also an independent risk for poorer prognosis and increased the risk of intraoperative complications [19]. In the current study the overall 5-year survival was 37.5% in patients without major intraoperative complications and 40.2% in patients with major intraoperative complications which underlines that the correlation between intraoperative complications and long-term survival is controversial. The effect of intraoperative complications to long-term survival is somehow multifactorial depending on patient, cancer and also surgery related factors [19, 33]. Nowadays there are two classification systems, the CLASSIC [25] and ClassIntra [7] classifications, which are both designed to standardize the reporting of intraoperative complications, but they differ in structure, validation, and intended use. However, standardizing the reporting of intraoperative complications could improve comparability across studies and enhance understanding of the impact of intraoperative complications on short- and long-term outcomes after gastric cancer surgery.

## Conclusion

In conclusion, major intraoperative complications did not seem to increase the risk of major postoperative complications, but the risk of infectious postoperative complications might be higher in patients with major intraoperative complications. The rate of 90-day mortality was non-significantly higher in patients with major intraoperative complications. Major intraoperative complications did not have effect on 5-year survival after gastric cancer surgery in this population-based nationwide study.

## Data Availability

The study data cannot be made publicly available due to laws and regulations. The data are available upon reasonable request from JHK, given that the registry holders’ permissions to use the data are obtained.
